# Editorial: Phonological Representations and Mismatch Negativity Asymmetries

**DOI:** 10.3389/fnhum.2022.916074

**Published:** 2022-06-01

**Authors:** Arild Hestvik, Mathias Scharinger, Valerie L. Shafer, Aditi Lahiri

**Affiliations:** ^1^Department of Linguistics and Cognitive Science, University of Delaware, Newark, DE, United States; ^2^Research Group Phonetics, Philipps-University of Marburg, Marburg, Germany; ^3^Center of Mind, Brain and Behavior, Universities of Gießen and Marburg, Marburg, Germany; ^4^PhD Program in Speech-Language-Hearing Sciences, The Graduate Center, City University of New York, New York, NY, United States; ^5^Faculty of Linguistics, Philology and Phonetics, University of Oxford, Oxford, United Kingdom

**Keywords:** phoneme, MMN, asymmetry, underspecification, abstractness

In this Topic, we attempt to refresh the discussion on asymmetries in speech sound representation and speech sound access, measured by means of the Mismatch Negativity (MMN, Näätänen and Alho, 1997). We collected contributions using a diversity of different approaches, in different languages and with different foci of their methodological specifications. All contributions share the quest for explaining asymmetries in speech perception, and all contributions attempt to provide interpretations couched in phonological theories. The collection of these studies shows that not only are asymmetries real and neurobiologically plausible, but rather abundant in the neurophysiology of human speech perception. The challenge that future contributions are faced with consist of cases in which phonological representations seem to modulate the MMN over and above acoustic effects. Cross-linguistic studies can address these interactions, since the acoustic effects will be stable (as the human auditory system is assumed to be uniform across cultures), whereas phonological systems vary across languages. The studies in this special issue provide cross-linguistic evidence that illustrates this interaction.

Riedinger et al. examines a larger set of vowel contrasts than usually covered in MMN studies. A total of 5 contrasts, embedded in existing German nouns, was presented in both directions (/i:/–/e:/, /e:/–/a:/, /y:/–/u:/, /i:/–/u:/, and /i:/–/a:/). Importantly, both MMN amplitudes as well as reaction times from an additional active oddball paradigm without neural measures showed asymmetries which were explicable within the FUL framework (Lahiri and Reetz, 2002), but less so in the NRV-framework (Polka and Bohn, 2003; Masapollo et al., 2017). A certain amount of variation was also explained by the auditory property of “perceived loudness” which in some cases resulted in a response pattern neither compatible with FUL nor with NRV. These results indicate that it is important to consider variables beyond those that are typically addressed in studies using MMN.

Yu and Shafer ask whether neural processing of the spectral differences between the English lax vowels /I/ and /ε/ was compatible with an underspecification account in which [-high] and [-low] are the underspecified values. Their findings were compatible with the underspecification approach, but also with other models, including the NRV model and Patricia Kuhl's Native Language Magnet model (Kuhl, 1991). In addition, they observed an effect of order of condition presentation (which stimulus first served as the standard in blocks that alternated which stimulus functioned as standard and deviant) and caution that non-linguistic factors also need to be considered as possible sources of speech processing asymmetries (see also Fitzgerald et al., 2018).

Meng et al. examine the predictions of FUL when a phonological assimilation process interacts with underspecification. Mandarin sibilants /s/ and /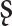/ are distinguished by tongue height features: /s/ being [LOW] and /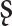/ underspecified. Consequently, height of the following vowel can spread to /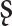/ which correctly predicted asymmetric MMNs in the context of the high vowel /u/ (i.e., /su/[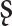
u] > /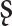
u/[su]) but symmetric MMNs for the low vowel /a/. This paper adds to the body of literature providing evidence for grammatically conditioned MMN asymmetry.

Fu and Monahan takes a novel approach in testing the nature of phonological representations by allowing inter-category variation in manner features (e.g., stops, fricatives) while testing the status of the place feature [retroflex], that does not vary across the set. They observed an MMN only when the standard stimuli belonged to the retroflex category, showing that a single shared phonological feature characterizing a natural class of standards was used as an abstract memory trace and generated an MMN, as well as providing evidence for underspecification of this feature in Mandarin.

de Rue et al. replicates the finding from German (Eulitz and Lahiri, 2004), showing an asymmetry supporting underspecification of the [coronal] place feature. However, the study also found an asymmetry for a contrast of labiality not seen for this mid-front vowel contrast in the previous study of German vowels. This partial replication reiterates the need for further studies of neuro-phonological representation extending to more languages.

Zora et al. address how Swedish words altered by phonetic assimilation activate their underlying lexical forms and examine whether neural processing is modulated by pre-lexical or lexical information. They found some support for the influence of pre-lexical processing, in observing a significant MMN only for a stimulus change to an unassimilated form (coda /m/ in the context of onset /n/ or coda /n/ in the context of onset /m/), but attestation of the assimilation pattern (that is, coda /n/ -> /m/ in the context of /m/), did not modulate the MMN. The findings are evaluated from the perspective of competing accounts of how phonetically altered surface forms activate their underlying lexical representations.

Polka et al. examine MMN asymmetries from the perspective of the Natural Referent Vowel theory and the Native Language Magnet theory, where MMN asymmetries would result from contrasts between prototypical and non-prototypical or focal/non-focal vowels. No MMN asymmetries were observed; instead, they found differences in the frequency domain that suggested processing advantages for focal vs. non-focal vowels and interpreted this to suggest increased cognitive demand presented by non-focal (atypical) vowels.

Nudga et al. examine MMNs for two phonemic distinctions in Czech: vowel quality [(a) vs. (e)], and vowel length [(e) vs. (e:)]. In order test the hypothesis that asymmetries only arise with linguistic representations, they also tested the same spectral and durational properties in non-speech stimuli. An asymmetry was observed only in the linguistic condition, indicating that the asymmetry does not arise from acoustic differences alone, but is conditioned by phonological features.

Cummings et al. examine asymmetry predictions for the contrast between /w/ and /r/ under the assumption that /w/ is underspecified for [CONSONANTAL]. They observed smaller MMN for /r/ than for /w/, consistent with the predictions of FUL. In a second contribution, they examine the contrast between /ba/ and /da/ in typically developing vs. language disordered children and found that neither group exhibited the adult-like asymmetries predicted by FUL. This suggests that underspecification becomes part of the grammatical system through language development.

## Author Contributions

All authors listed have made a substantial, direct, and intellectual contribution to the work and approved it for publication.

## Conflict of Interest

The authors declare that the research was conducted in the absence of any commercial or financial relationships that could be construed as a potential conflict of interest.

## Publisher's Note

All claims expressed in this article are solely those of the authors and do not necessarily represent those of their affiliated organizations, or those of the publisher, the editors and the reviewers. Any product that may be evaluated in this article, or claim that may be made by its manufacturer, is not guaranteed or endorsed by the publisher.

## References

[B1] Eulitz C. Lahiri A. (2004). Neurobiological evidence for abstract phonological representations in the mental lexicon during speech recognition. J. Cogn. Neurosci. 16, 577–583. 10.1162/089892904323057308 15185677

[B2] Fitzgerald K. Provost A. Todd J. (2018). First-impression bias effects on mismatch negativity to auditory spatial deviants. Psychophysiology. 55. 10.1111/psyp.13013 28972671

[B3] Kuhl P. K. (1991). Human adults and human infants show a “perceptual magnet effect” for the for the prototypes of speech categories, monkeys do not. Percept. Psychophys. 50, 93–107. 1945741 10.3758/bf03212211

[B4] Lahiri A. Reetz H. (2002). Underspecified recognition, in Laboratory Phonology VII, eds Gussenhoven C. Warner N. (Berlin: Mouton de Gruyter), 637–677.

[B5] Masapollo M. Polka L. Ménard L. (2017). A universal bias in adult vowel perception - By ear or by eye. Cognition. 166, 358–370. 10.1016/j.cognition.2017.06.001 28601721

[B6] Näätänen R. Alho K. (1997). Mismatch negativity (MMN) - the measure for central sound representation accuracy. Audiol. Neurootol. 2, 341–353. 10.1159/000259255 9390839

[B7] Polka L. Bohn O. S. (2003). Asymmetries in vowel perception. Speech Commun. 41, 221–231. 10.1016/S0167-6393(02)00105-X

